# Complete Genome Sequence of Collection Strain Acinetobacter baumannii ATCC BAA-1790, Used as a Model To Study the Antibiotic Resistance Reversion Induced by Iodine-Containing Complexes

**DOI:** 10.1128/MRA.01467-19

**Published:** 2020-01-16

**Authors:** Ilya S. Korotetskiy, Monique Joubert, Sade M. Magabotha, Ardak B. Jumagaziyeva, Sergey V. Shilov, Natalya A. Suldina, Sabina T. Kenesheva, Anna Yssel, Oleg N. Reva, Aleksandr I. Ilin

**Affiliations:** a Scientific Center for Anti-Infectious Drugs (SCAID), Almaty, Kazakhstan; b Centre for Bioinformatics and Computational Biology (CBCB), Department of Biochemistry, Genetics and Microbiology, University of Pretoria, Pretoria, South Africa; c Faculty of Biology and Biotechnology, al-Farabi Kazakh National University, Almaty, Kazakhstan; d Centre for Microbial Ecology and Genomics (CMEG), Department of Biochemistry, Genetics and Microbiology, University of Pretoria, Pretoria, South Africa; Queens College

## Abstract

The strain Acinetobacter baumannii ATCC BAA-1790 was sequenced as a model for nosocomial multidrug-resistant infections. Long-read PacBio sequencing revealed a circular chromosome of 3,963,235 bp with two horizontally transferred genomic islands and a 67,023-bp plasmid. Multiple antibiotic resistance genes and genome methylation patterns were identified.

## ANNOUNCEMENT

Acinetobacter baumannii is a common nosocomial pathogen harboring resistance to a wide variety of antibiotics. The multidrug-resistant strain A. baumannii ATCC BAA-1790 was isolated from sputum in 2008 in Washington, DC. In this study, it is used as a model organism to investigate genomic and population changes under the effect of a new drug, FS-1, which induces the reversion of multidrug-resistant bacteria to their antibiotic-sensitive phenotypes (1, 2).

**FIG 1:**
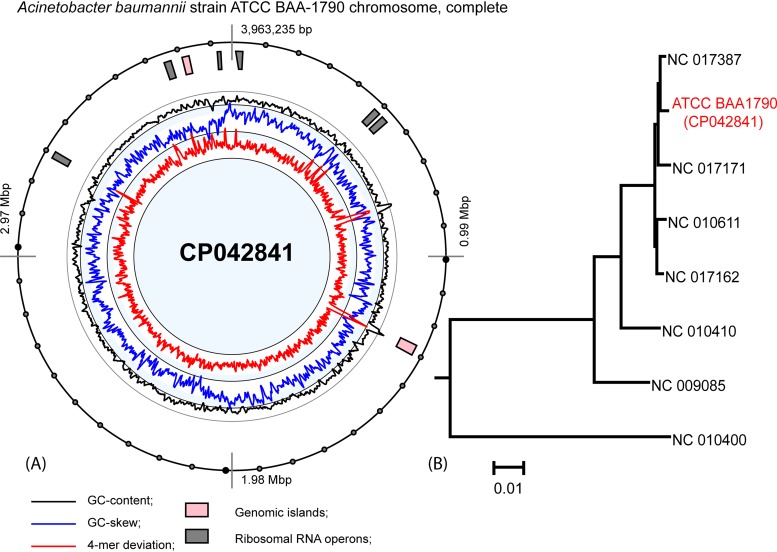
(A) The atlas view generated by the program SeqWord Genomic Island Sniffer (13) shows a circular map of the chromosome with predicted genomic islands, rRNA gene (*rrn*) clusters, and histograms of the GC content, GC skew, and tetranucleotide pattern deviations calculated in an 8-kbp sliding window stepping 2 kbp. (B) The neighbor-joining phylogenetic tree shows relations of the sequenced strain BAA-1790 with reference genomes of A. baumannii.

The strain was obtained from ATCC (https://www.lgcstandards-atcc.org/) and cultivated in Mueller-Hinton broth (HiMedia, India) without antibiotics at 37°C with shaking. DNA was extracted from the overnight culture using the cetyltrimethylammonium bromide (CTAB) protocol (3). Sequencing was performed at Macrogen according to the SMRTbell preparation guide for the PacBio RS II platform, resulting in the generation of 326,117 reads with an average length of 10,000 bp (*N*_50_, 8,662 bp). DNA reads were quality controlled and trimmed using the UGENE v1.32.0 raw DNA-Seq processing pipeline with default parameters (4). Default parameters were used for all subsequent analyses. *De novo* assembly of the complete genome was done using the polished_falcon_fat pipeline, available from the SMRT Link v5.0.1 software (5). Two ungapped contigs with a coverage of 100× were obtained and corresponded to a 3,963,235-bp chromosome and a 67,023-bp plasmid with average GC contents of 39.16% and 33.38%, respectively. The RAST annotation server (6) was used to automatically generate annotations for the genome, followed by manual correction. Genomic islands present on the chromosome (Fig. 1A) contributed to the antibiotic resistance of the strain, with carbapenem-hydrolyzing class D beta-lactamase OXA-23, a Sul1 sulfonamide resistance protein, and an integrase insert comprising *aadA1* (aminoglycoside 3′-adenyltransferase) and *aadC1* (aminoglycoside *N*(3′)-acetyltransferase type I), rendering the strains resistant to aminoglycosides (7). Multiple genes for drug efflux pumps and beta-lactamases of the OXA-23, OXA-82, and ADC-25 families providing resistance to cephalosporins and penams were identified using the Resistance Gene Identifier (RGI) server (8). Comparing the sequences of 2,588 orthologous proteins identified by OrthoFinder (9), which were shared by sequenced genomes of A. baumannii, showed that the closest phylogenetic relationship of strain BAA-1790 to A. baumannii TCDC-AB0715 (Fig. 1B) produced an increasing trend of carbapenem and fluoroquinolone resistance (10). The plasmid identified in strain BAA-1790 is typical for many A. baumannii isolates. It shows more than 99% DNA sequence identity with the plasmids pMAL-2 (KX230794) and FDAARGOS_493 (CP033857). The plasmid contains multiple genes associated with plasmid replication and conjugation and the *telA* toxic anion resistance gene.

The SMRT Link DNA modification pipeline, ds_modification_motif_analysis, was used for profiling of epigenetic modifications in the bacterial genome. Two major motifs, AGCNNNNGCT and TGGCCA, associated with methylation of the underlined adenine and cytosine residues, were discovered. Methylation of adenine at AGCNNNNGCT is caused by M.Aba7804III methyltransferase (11), and cytosine methylation at TGGCCA is associated with the activity of the BalI restriction-modification system, also common for A. baumannii (12).

### Data availability.

The genome is available from NCBI under the accession numbers CP042841 and CP042842 for the chromosome and plasmid sequences, respectively. The PacBio reads are available under the SRA numbers SRR10112456, SRR10112460, and SRR10112461. The BioProject accession number is PRJNA557366.
